# A randomized clinical prospective trial comparing split-dose picosulfate/ magnesium citrate and polyethylene glycol for colonoscopy preparation

**DOI:** 10.1371/journal.pone.0211136

**Published:** 2019-03-28

**Authors:** Alaa Rostom, Catherine Dube, Kirles Bishay, Lilia Antonova, Steven J. Heitman, Robert Hilsden

**Affiliations:** 1 Department of Medicine and Ottawa Hospital Research Institute, University of Ottawa, Ottawa, Canada; 2 Department of Medicine and Community Health Sciences, University of Calgary, Calgary, Canada; University Hospital Llandough, UNITED KINGDOM

## Abstract

**Background:**

Colonoscopy remains the gold standard for the investigation of abnormalities within the colon. However, its success is highly dependent on the quality of bowel preparation. The objective of this study was to compare the bowel preparation efficacy of picosulfate/magnesium citrate (PMC) vs polyethylene glycol (PEG) in a one-day vs two-day split dose regimen.

**Methods:**

A prospective, randomized, controlled trial was conducted at the Forzani & MacPhail Colon Cancer Screening Centre in Calgary, Canada. 171 colonoscopy outpatients were randomized to split-dose PMC or PEG lavage as well as into one-day split or two-day split regimens in blocks of eight. Bowel preparation quality was recorded in a blinded manner by the endoscopist using the Ottawa Bowel Preparation Scale (OBPS) prior to washing or suctioning. The scale results were analyzed using a two-factor analysis of variance.

**Results:**

141 patients received complete colonoscopies (PMC-71; PEG-70). PEG was found to be superior to PMC (mean OBPS: 4.14 ± 2.64 vs 5.11 ± 3.44, p = 0.019), when adjusted for administration regimen, leading to significantly more adequate bowel preparations (79.7% vs 59.7%, p = 0.007). A two-day split dose was superior to a one-day split dose regimen (mean OBPS: 3.68± 2.82 vs 5.69 ± 3.06, p<0.001). Two-day split dosing also resulted in a better right colon cleanliness score (right bowel OBPS 1.27±0.11 vs 2.10±0.12 for one-day split, P<0.001).

**Conclusions:**

Optimal bowel preparation was achieved with the use of PEG lavage when administered in a two-day split dose regimen. This trial is registered with ClinicalTrials.gov under identifier NCT01415687.

## Introduction

Colonoscopy is the gold standard for the investigation of abnormalities within the colon and is an integral part of all colorectal cancer screening programs. The ability of colonoscopy to detect high-risk lesions is greatly dependent on the quality of pre-colonoscopy bowel preparation. Poor bowel preparation results in longer procedures, need for repeat colonoscopy and missed lesions [1] and has been observed in as many as 25% of cases.[1–3] One level of consideration in selecting an agent relates to the physico-chemical properties of the agent itself, including solution tonicity, safety profile, as well as the volume required to produce adequate bowel preparation. Patient factors, such as comorbidity burden and cost of various preps, also represent important considerations in selecting an agent. Lastly, a decision should be based on published evidence of the agent’s efficacy as a colonic lavage solution. Based on these factors, PEG and PMC have emerged as two of the most commonly used agents clinically.

Since its introduction in 1980 by Davis *et al*, polyethylene glycol (PEG) remains one of the safest and most efficacious bowel regimens available.[4] PEG is a non-absorbable, large polymer that remains in the gut lumen resulting in a lavage effect through osmosis.[5] It is a balanced isotonic electrolyte solution that minimizes fluid shifts across the colon, as well as the potential of mucosal damage. However, due to its large volume and taste particularity, its effectiveness may be influenced by problems with patient tolerance and regimen compliance.[6]

Picosulfate/magnesium citrate (PMC) is another purgative which offers an alternative to the large volume required with PEG. PMC is generally made up of two components: Sodium Picosulfate, a prodrug that is metabolized by the colonic flora into an active metabolite stimulating peristalsis, as well as magnesium oxide and citric acid, which react to create magnesium citrate, inducing catharsis and adding an osmotic effect within the GI tract.[7] Due to its hyperosmotic nature, PMC does appear to be more likely to result in fluid shift, dehydration, electrolyte disturbances, as well as orthostatic hypotension.[5,8] Therefore, it is used with caution in children, the elderly, patients with renal insufficiency, or patients with known congestive heart failure. The smaller volume of PMC necessary for effective bowel cleansing generally makes it more palatable for patients,[9] though sufficient oral fluid intake is required to guard against dehydration.

Beyond the choice of preparation agent, the timing of agent administration has also been observed to be a significant factor in achieving high quality colonic cleansing. Specifically, there is strong evidence that administration of the preparation agent in a split dose is superior to administration in a single dose in terms of patient convenience, palatability, improved bowel preparation and increased polyp detection.[10–15]

The objective of this study was to compare the efficacy of one- and two-day split dosing of PMC and PEG for preparing the bowel for colonoscopy, as assessed by the Ottawa Bowel Preparation Scale (OBPS), a validated scale for the evaluation of bowel preparation quality.[16] Specifically, the OBPS was chosen as it utilizes scoring performed prior to suction and wash within the colon, thus providing the best measurement of preparation regimen efficacy. The impact of both preparation agent and intake timing on the quality of bowel preparation in the right colon was assessed.

## Patients/Materials and methods

This trial was approved by the University of Calgary institutional ethics board on April 21, 2011 and registered with ClinicalTrials.gov under identifier NCT01415687. A small delay in registration was caused by an administrative error. No changes in the protocol occurred during this period. This study has no other related past or ongoing trials.

Patients aged 18 to 74 years who were referred to the Forzani & MacPhail Colon Cancer Screening Centre (CCSC) in Calgary, Alberta, Canada between May 2011 and December 2011 for outpatient colonoscopy, were considered for inclusion in the trial. In keeping with CCSC’s practice, indications for colonoscopy were exclusively screening-related, and included average-risk screening, increased-risk screening (patients with one or more first degree relative[s] with colorectal cancer [CRC] or colorectal adenomas, hereditary CRC syndromes), adenoma surveillance or positive fecal occult blood test. A nurse clinician recruited patients to the trial at the time of their initial, pre-procedural, clinical assessment.

Exclusion criteria for enrollment into the study were: a history of acute coronary syndrome; congestive heart failure; unstable angina; known or suspected renal failure; ascites; megacolon; known or suspected bowel obstruction; or other significant comorbidities. Patients were also excluded if they previously had a partial or subtotal colectomy or if the patient reported symptoms of diarrhea.

Enrollment of participants was performed by a central study coordinator. Computer-generated randomization with blocks of eight was used to ensure equal distribution into the one- or two-day split dosing. Consecutively numbered sealed envelopes were used to maintain allocation concealment. The allocation ratio was 1:1. Endoscopists and investigators were blinded to the allocation groups.

Patients were randomly allocated to one of two groups consisting of either PEG or PMC with preparation taken either in two doses the day prior to the procedure (one-day split group) or with one dose the evening prior to the procedure and the second dose in the morning of the procedure (two-day split group). The one-day split group patients were booked for their procedure before 10 AM to minimize the delay between preparation dose and endoscopy. Patients in this group were instructed to take half of their preparation (either 2L of PEG or one sachet of PMC) between noon and 4:00 pm the day prior to colonoscopy and the second half at 8 PM the same evening. The two-day split group patients were scheduled for procedures scheduled after 10 AM. They were instructed to take the first half of their preparation solution at 8 PM the night prior to colonoscopy and the second dose 5 hours before the procedure.

A nurse study coordinator assigned patients to their group and instructed them on the proper use of their assigned bowel preparation method. Patients were told to start a low residue diet four days prior to the colonoscopy and received handouts listing foods to consume and avoid. Patients were instructed to take a light breakfast on the morning before the procedure, followed by clear fluids thereafter. Patient concerns or questions regarding the preparation were directed to the study coordinator to avoid unblinding the endoscopist.

### Data collection

The OBPS[16] was used as an objective and validated means of assessing bowel cleanliness (Table 1). This scale involves assigning a score between 0–4 to each of the right, mid and rectosigmoid colon and a global fluid score between 0–2 for a total score out of 14. Segment scores range from 0, indicating an excellent preparation with superior mucosal visualization with minimal stool or fluid, to a score of 4, which is assigned in instances of inadequate preparation when there is frank stool blocking visualization of the colonic mucosa. Similarly, a fluid score of 0 is assigned when there is minimal fluid in the colon and a score of 2 is assigned when there is copious fluid. Total scores close to 0 suggest excellent preparation while scores close to 14 represent completely inadequate or unprepared colons. In accordance with previous studies, a total OBPS score of 6 or lower is defined as adequate bowel visualization.[10,16] The OBPS is applied prior to irrigation and suction, thus providing a direct evaluation of bowel regime effectiveness.

A simplified bowel preparation scale was also recorded in the course of the trial, but was outside of the scope of this study.

**Table 1:** Ottawa bowel preparation scale.

Preparation Quality	Score
*Assessment of Right*, *Mid and Rectosigmoid Colon*	
No Liquid	0
Minimal Liquid, No Suction Needed	1
Suction Needed	2
Suction and Wash Needed	3
Solid Stool, Not Washable	4
*Assessment of Entire Colon*	
Global Quantity of Fluid	0–2

Operators were trained on the use of OBPS prior to initiation of the trial. Scores were recorded on a standardized form by endoscopists, at the time of colonoscopy, before any attempt to improve visualization (i.e. wash or suction) was initiated.

### Statistical analyses

Statistical analysis was performed using SPSS V20, IBM Corporation and Stata 12, StataCorp LLC. Baseline characteristics were collected for each patient for the performance of descriptive statistics. Overall and segmental OBPS scoring is approximately normally distributed, thus two-group ANOVA was used for group differences with regard to preparation solution, while accounting for timing (i.e. one-vs two-day split) (two-sided, 0.05 significance level). Likewise, two-group ANOVA was used for group differences with regard to timing, while accounting for preparation solution. The Chi-square test was used to compare adequacy of bowel visualization between the bowel preparation groups.

### Sample size calculation

For the study to have 80% power to detect an effect size of 1.5-point difference in total OBPS between preparation reagent groups or timing groups, with a two-tailed alpha at 0.05, a total of 144 patients were required. A difference of 1.5 on OBPS is considered significant, based on our findings from previous studies.[10,16] Based on experience from prior studies, an additional 20% (n = 29) of patients were added to the total sample size, to account for drop-outs and incomplete colonoscopies.

## Results

**Fig 1:**
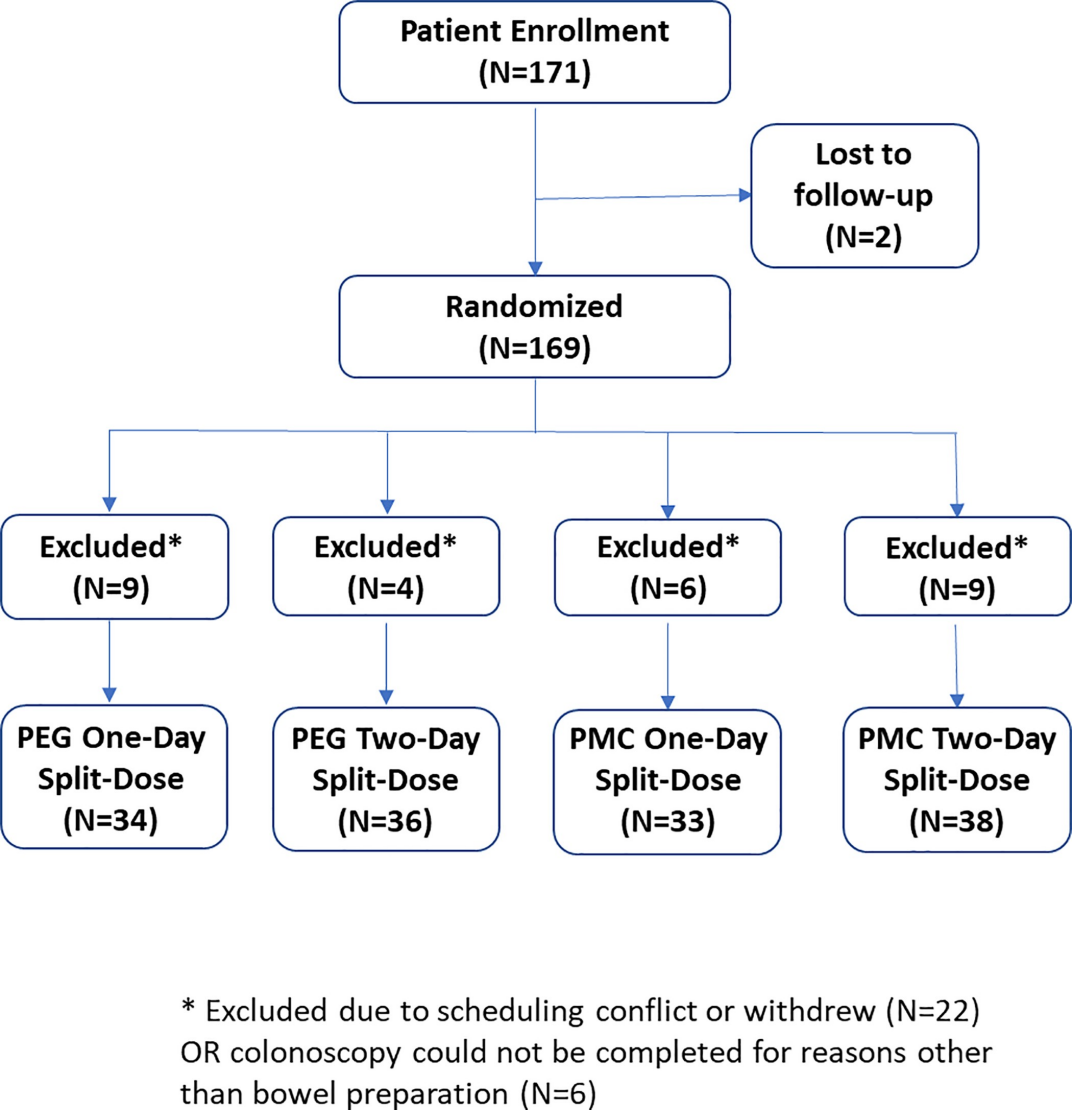
Study flow diagram.

In total, between May 2011 and December 2011, 171 patients were enrolled in the trial (Fig 1). Of these, 141 underwent a complete colonoscopy. Colonoscopy could not be completed for reasons other than poor bowel preparation in 6 patients and 22 patients either withdrew from the trial or had scheduling conflicts for colonoscopy post-randomization. The distribution of patients that did not complete the study was similar between treatment groups, as shown in Fig 1. No adverse events occurred during the trial that resulted in discontinuation. A total of 70 patients received PEG and 71 received PMC. Table 2 shows the baseline characteristics of the two groups. PMC and PEG groups were well matched in terms of both age (57.6[95% CI:55.8,59.4] vs 56.4[95%CI:55.0,57.8]), P = 0.395) and sex (percent female: 47.8% vs 51.4%, P = 0.539). In the PEG group, 34(48%) patients received a one-day split regimen and 36(52%) patients received a two-day split regimen. In the PMC group, 33(46%) patients received a one-day split regimen and 38(54%) received a two-day split regimen. Patient compliance to their assigned regimen exceeded 95% in all groups, due to the personalized nurse-led patient training provided at the screening center prior to colonoscopy preparation.

**Table 2:** Baseline patient characteristics.

Characteristic	PEG (N = 70)	PMC (N = 71)	P
**Female sex–no (%)**	36 (51.4)	34 (47.8)	0.539
**Mean age–years**	56.4 (95%CI:55.0,57.8)	57.6 (95%CI:55.8,59.4)	0.395
**Age group in years**	**Number (%)**	**Number (%)**	0.169
<50	6(42.8)	8(57.1)	
50–59	39(58.2)	28(41.8)	
60–69	24(44.4)	30(55.5)	
70–75	1(16.7)	5(83.3)	

**Fig 2:**
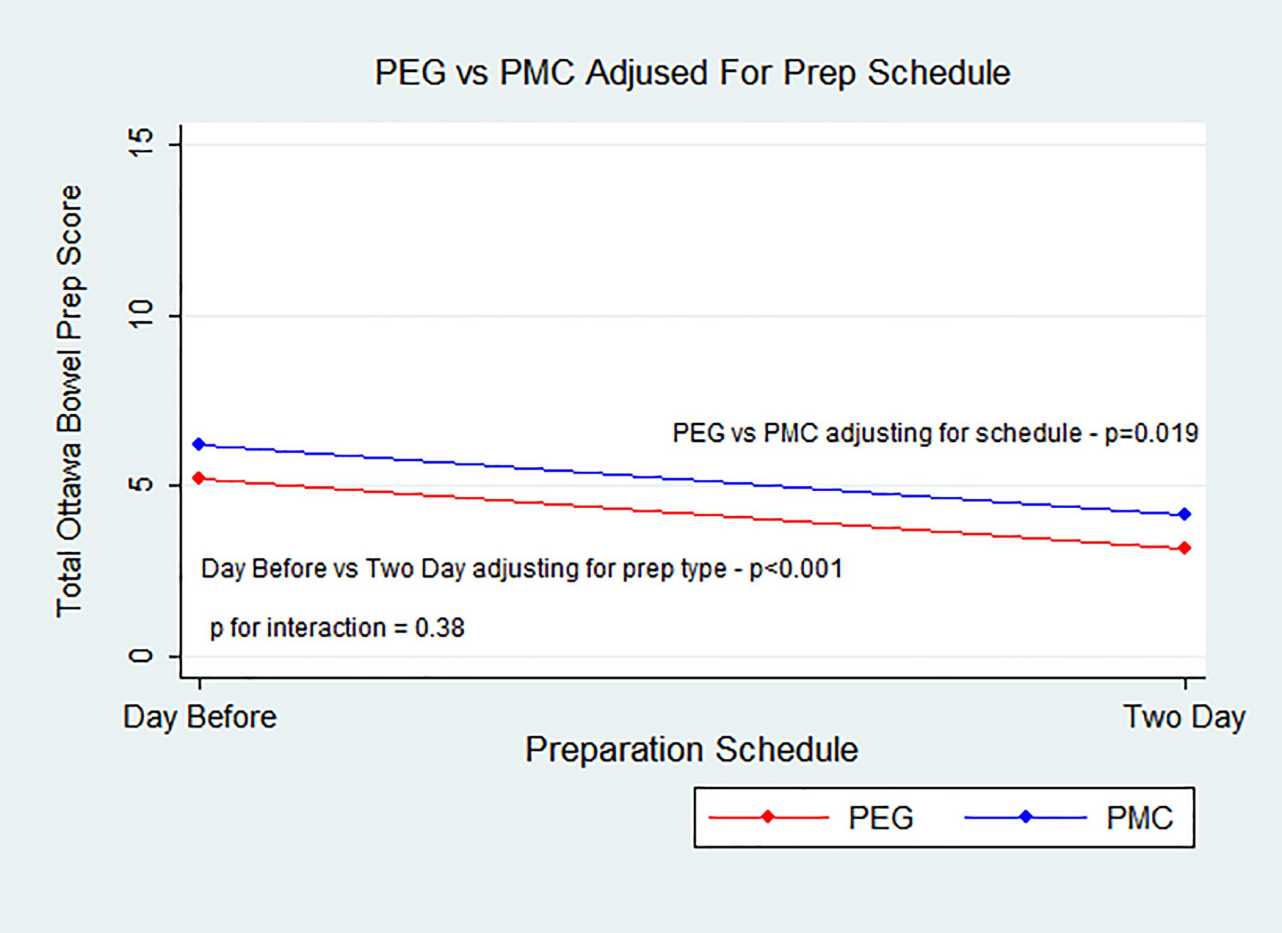
Mean OBPS for PMC and PEG, adjusting for timing of administration. PEG is superior with a lower mean score of 4.14±2.64 compared to PMC, which has a mean score of 5.11±3.44 (P = 0.019).

**Fig 3:**
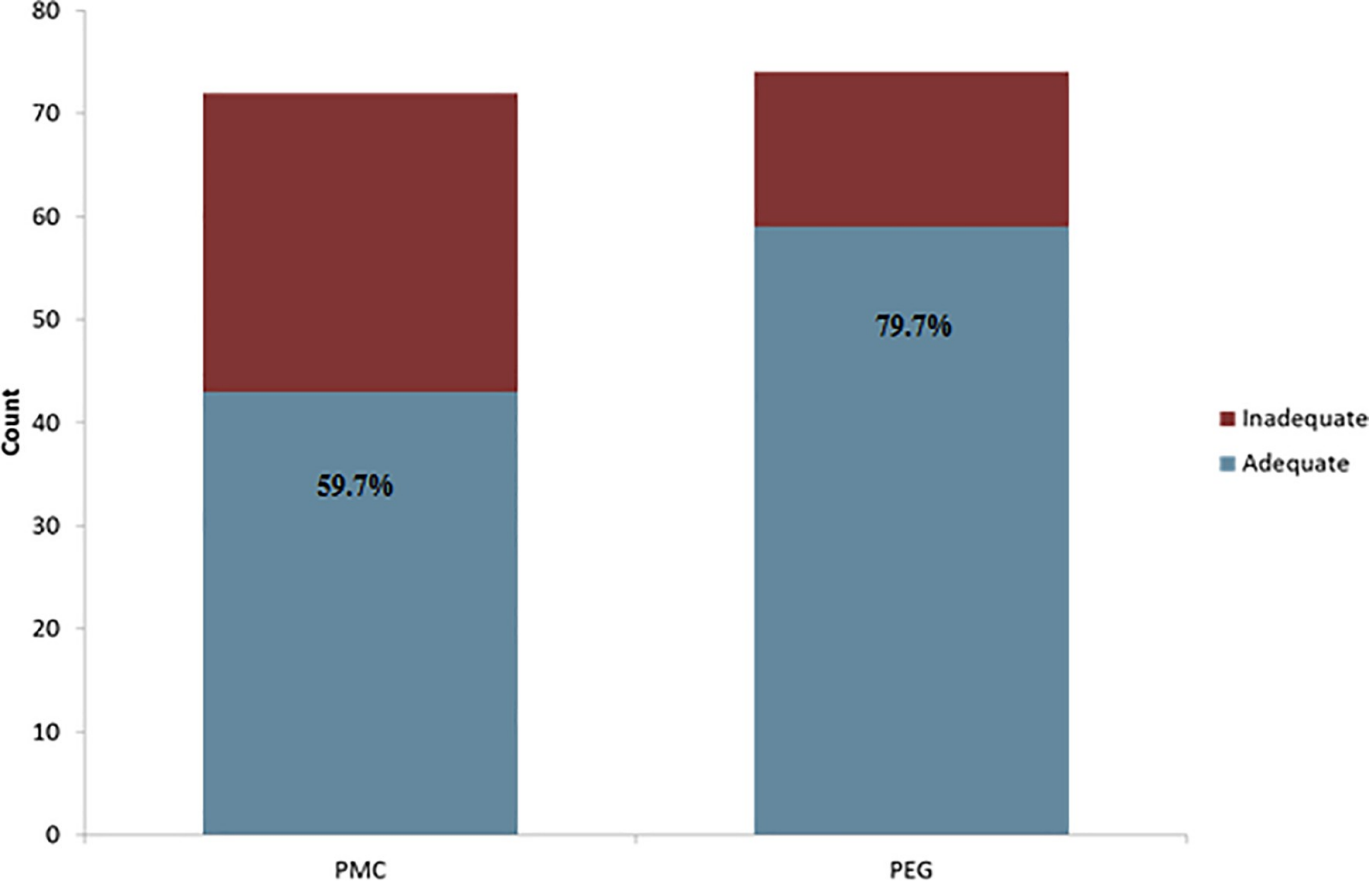
Proportion of patients with adequate bowel preparation defined as an OBPS less than 6. More adequate preparations occur using PEG compared to PMC (79.7% vs. 59.7%, P = 0.007).

Bowel preparation in the PEG group was superior to that of the PMC group, when accounting for split dose timing (mean OBPS: 4.14±2.64 vs. 5.11±3.44, P = 0.019, Fig 2). The rate of adequate bowel preparation defined as an OBPS less than 6 was also superior among patients receiving PEG compared to PMC (79.7%, vs 59.7% P = 0.007, Fig 3). Bowel preparation in the two-day split group had a better (lower) mean OBPS compared to the one-day group, regardless of bowel preparation type (3.68±2.82 vs. 5.69±3.06, P<0.001, Fig 2).

There was no statistical interaction between preparation type and timing of preparation administration (one-day/two-day) (p = 0.384, Fig 2).

**Fig 4:**
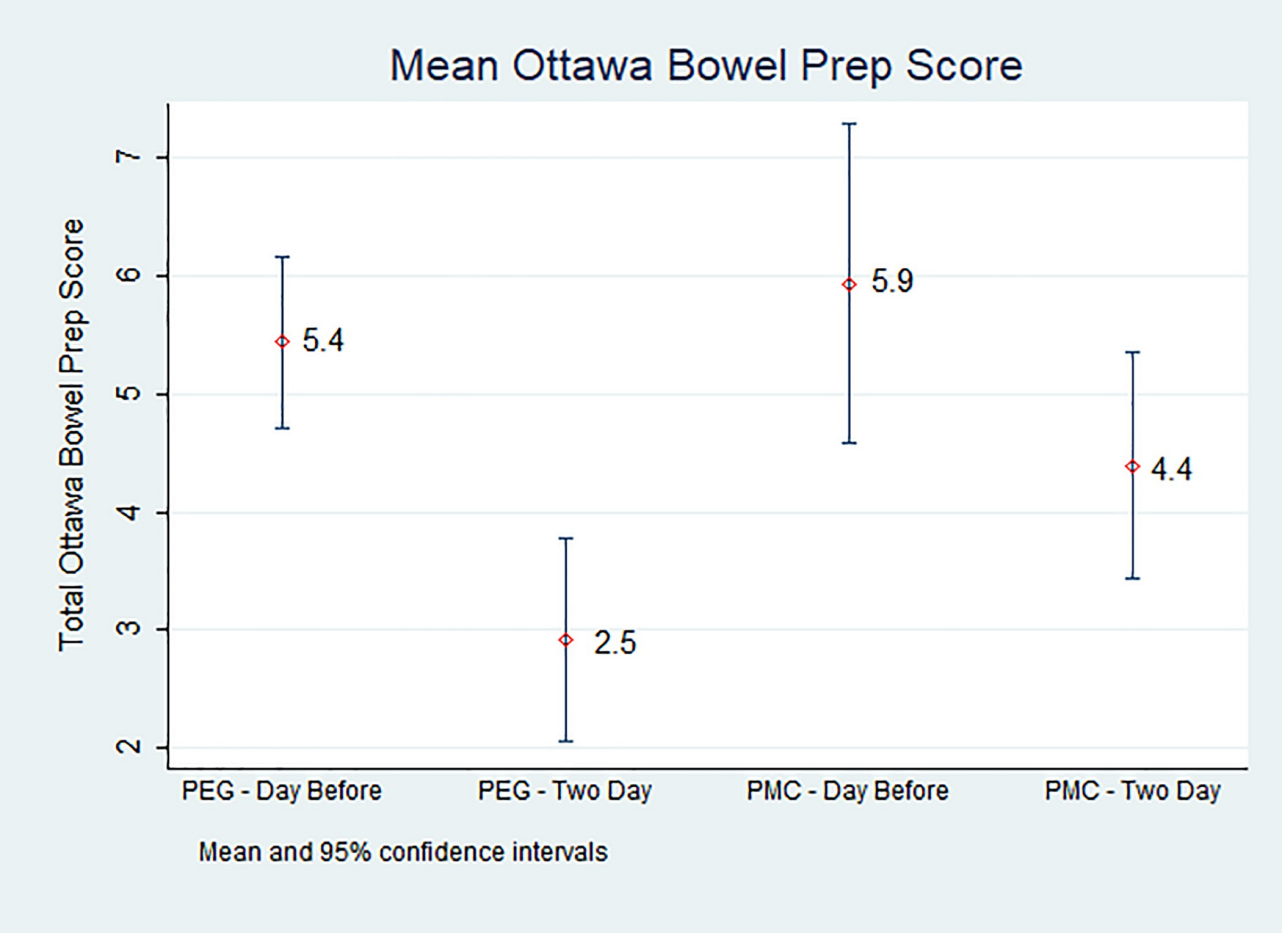
Unadjusted mean OBPS by preparation regimen and timing with 95% confidence intervals. The PEG two-day split group resulted in the best bowel preparation (OBPS 2.92; 95% CI:2.1–3.8), followed by PMC two day split (OBPS 4.39; 95% CI: 3.4–5.4). PMC and PEG one day-split groups both had higher OBPS scores (5.94; 95% CI: 4.6–7.3 and 5.44; 95% CI: 4.7–6.2, respectively) (Fig 4).

Preparation quality in the right colon was specifically assessed using the segment-specific OBPS (score out of 4). The utilization of PEG vs. PMC did not have any effect on cleanliness of the right colon, when corrected for administration regimen (OBPS for right colon 1.60 vs 1.76, P = 0.33). However, timing of administration demonstrated a strong effect on cleanliness in favor of the two-day split group, when adjusted for reagent (OBPS for right colon 1.27 vs 2.10, P<0.001).

## Discussion

In this trial, we compared the bowel-cleansing effectiveness of PEG and PMC, as well as that of day-before versus two-day preparation regimen. Our results demonstrate that PEG is superior to PMC for lavage of the bowel, after adjustment for one-day vs two-day split dosing. Previous findings indicate that the difference of nearly 1 point on the OBPS produced by PEG administration is likely to be clinically significant.[10,16] Furthermore, we found that a two-day preparation regimen produces superior bowel cleansing, as compared to a one-day preparation regimen. The difference produced by preparation timing was 2 points on the OBPS. Our results indicate that a change to a two-day split dose regimen can have a greater impact on colon cleansing than the choice of preparation agent. Optimal OBPS was obtained when PEG was utilized in a two-day preparation regimen. Segment scores did not differ between the preparation agents. However, when assessing the right colon, there was a strong effect in favor of two-day bowel preparation.

Several studies have looked at different bowel regiments for optimizing colonoscopy examinations, though the timing of administration in these trials has been inconsistent. A recent meta-analysis performed by Jin et al., comparing PMC and PEG, revealed non-inferiority of PMC, though there was a trend in favor of PEG.[17] As our study results indicate that the timing of bowel preparation administration has a strong effect on the readiness of the bowel for colonoscopy, the difference in outcome in the Jin et al study, as compared to the current trial, may be a product of the heterogeneity of administration regimens included in the meta-analysis. This point is highlighted by another recent meta-analysis of 47 trials that compared split-dose to day-before bowel cleansing.[18] Martel *et al*. demonstrated that the two-day regimen is superior for achieving adequate bowel preparation for colonoscopy. Further, subgroup analyses revealed superiority of two-day preparation for both PEG and PICO, consistent with the results of our study.

The superiority of two-day split dose administration can be explained by the timespan between ingestion of the last dose of the preparation and the time of procedure. Previous studies that have explored preparation-to-colonoscopy time intervals have consistently found a shorter time span, between 3 to 7 hours, to be ideal for optimizing bowel preparation.[19–22] It is likely that the shorter timespan between ingestion of preparation agent and procedure minimizes the amount of time for bile and fluid to be secreted and accumulate in the colon.[21] Therefore, regardless of preparation agent used and time of the procedure, a dose of preparation should be administered during the above time window to optimize colonoscopy preparation. For morning procedures, this may involve taking a dose in the early morning. While there may be reluctance on the part of both clinicians and patients to do this, education strategies should be explored to convey the observed improved visualization and consequent reduction in the need for repeat procedures.

Several large observational studies have demonstrated that colonoscopy has minimal to no protective effect on the incidence of death from right-sided CRC.[23–25] The sessile serrated pathway of colorectal carcinogenesis has since been recognized as the cause of sporadic, predominantly right-sided neoplasia, characterized by biological, morphological and molecular features that are distinct from the adenoma-carcinoma sequence.[26–28] Serrated lesions are sessile or flat, similar in color to the surrounding mucosa, and often covered by a mucus cap making them more difficult to detect.[29] The ability to detect such lesions in the proximal colon is, therefore, particularly susceptible to inadequate bowel preparation.[30] The results of the present study suggest that a two-day bowel preparation could potentially increase serrated polyp detection rates in the right colon, though this merits further investigation.

A strength of this randomized controlled trial is that it was conducted in an outpatient colon cancer screening center with highly standardized operations and quality monitoring. This ensured consistent colonoscopy quality and high patient regimen compliance. Additionally, we used a validated evaluation scale that is highly appropriate to the study goals. The OBPS applies metrics prior to any operator attempt to cleanse the bowel and, thus, reflects the effect of bowel preparation agent alone. To ensure study validity, screening center endoscopists were trained on the OBPS prior to study commencement and were blinded to preparation regimen.

Finally, The Forzani & MacPhail Colon Cancer Screening Centre (CCSC) is a regional screening center that captures a socioeconomically and ethnically diverse population. Therefore, we expect that our findings would be generalizable to other patients 35 to 75 years that are eligible for colonoscopy.

A limitation of this study is that endoscopist adenoma detection rate (ADR) was not measured and should be taken into account in future studies in order to assess the direct effect of bowel preparation and timing on colonoscopy success. Patient reagent tolerability was not assessed in the course of the study. However, patient regimen compliance was found to be higher than 95%.

## Conclusions

The results of this trial demonstrate that bowel preparation quality can be optimized through the use of a two-day preparation regiment and the administration of a PEG based lavage. This suggests that even for early morning procedures, a second dose of preparation should be administered 5–6 hours prior to the procedure. It is imperative that patients are educated with regard to the need for good preparation, in order to optimize colonoscopy outcomes and reduce the need for repeat procedures.

## Supporting information

S1 File CONSORT checklist. (DOCX)

S2 File Study protocol. (PDF)
