# Supplementary material for: A randomized clinical prospective trial comparing split-dose picosulfate/ magnesium citrate and polyethylene glycol for colonoscopy preparation
Source: PLoS One. 2019 Mar 28;14(3):e0211136. doi: 10.1371/journal.pone.0211136 (PMC6438535; doi:10.1371/journal.pone.0211136)
Supplement: S2 File — (PDF) [file pone.0211136.s002.pdf]

APPLICATION FOR SCIENTIFIC, ADMINISTRATIVE & ETHICAL REVIEW OF CLINICAL TRIALS, HEALTH RESEARCH INVOLVING HUMAN SUBJECTS
("Human subject" includes human biosamples, tissue and genetic information as well as people)

UNIVERSITY OF CALGARY   calgary health region

NB: INTERIM APPLICATION FORM, March 2011

SECTION A- GENERAL INFORMATION

DATE RECEIVED BY: OMB/CHRO ________________ **GRANT ID #** ________________

1. PROTOCOL TITLE:

A Randomized Prospective Trial Comparing Pico-Salax (Magnesium Citrate) Plus Bisacodyl Versus Split Dose Polyethylene Glycol-Based Lavage In Preparation Of Patients For Colonoscopy

2. LOCAL PRINCIPAL INVESTIGATOR: Alaa Rostom

(Note: students, residents & fellow cannot be listed as PI)

FACULTY (RANK) and PRIMARY DEPARTMENT : Associate Professor – Medical Director Colon Cancer

PHONE: ________ FAX: ________ E-MAIL: arostom@ucalgary.ca

CO-INVESTIGATOR(s): Robert Hilsden – Director Research

(indicate student, resident, fellow with an)* Catherine Dube – Director – Quality Assurance

Research Coordinator/Assistant(s): Eleanor Rushton

Phone: 403-592-5087 Fax: 403-592-5066 E-Mail: ecrushto@ucalgary.ca

*********PLEASE CHECK THE FOLLOWING IF APPROPRIATE*********

☐ Masters/PhD Project ☐ Medical Student Project ☐ Undergraduate Project ☒ Resident or Fellow Project

3. ANTICPATED START DATE: April 2011

ANTICIPATED COMPLETION DATE: Six months from start date

ANTICIPATED NUMBER OF SUBJECTS (Local only): 150

4. TYPE OF RESEARCH: ☒ Clinical Trial ☐ Health Research ☐ Basic Science

5. LOCATION OF RESEARCH: *check all that apply*

Calgary Health Region

☐ FMC	☐ ACH	☐ Care in the Community
☐ PLC	☐ SAC	☐ Healthy Communities
☐ RVH	☐ CBH	☐ Other:

University of Calgary and other Sites

☐ UCMC	☐ Faculty of Kinesiology	☐ TBCC
☐ HMRC	☐ Faculty of Nursing	☐ Off-site Medical Office
☐ CHS	☒ Other: (Specify) Forzani & MacPhail Colon Cancer Screening Centre	

6. PROPRIETARY RIGHTS: *Please check one*

☒ The investigators can alter the protocol according to their judgment and have full rights to information derived from this research and publication of this information.

☐ This research is being done for a sponsor who controls the details of the protocol and the rights to the information gathered.

7. ***This serves as application for disclosure of health information to be used in research and I, on behalf of project personnel identified in this document, agree to the following*** **:**

a) to comply with the *Health Information Act* and all regulations under that Act [section 54(1)(a)(i)];

b) to comply with all conditions imposed by the CHR and the University of Calgary relating to the use, protection, disclosure, return or disposal of the health information [section 54(1)(a)(ii)];

c) to comply with all requirements of the CHR and the University of Calgary to provide safeguards, against the identification, direct, or indirect, of an individual who is the subject of the health information [section 54(1)(a)(iii)];

d) to use the health information only for the purpose of conducting the proposed research [section 54(1)(b)];

e) to not publish the health information in a form that could enable the identity of the subject of the health information [section 54(c)];

f) to not attempt to contact the subject of the health information except in accordance with the Act [section 54(d) and 55];

g) to allow the Custodian of health information access as prescribed by the Act [section 54(e)].

____

Local Principal Investigator's Signature

APPLICATION FOR SCIENTIFIC, ADMINISTRATIVE & ETHICAL REVIEW OF HUMAN SUBJECT CLINICAL TRIALS AND HUMAN SUBJECT HEALTH RESEARCH
("Human Subject" includes human biosamples, tissue and genetic information as well as people)

UNIVERSITY OF CALGARY   calgary health region

SECTION B – DEPARTMENT APPROVALS

Please obtain your Department Head's signature and signatures of all departments/divisions/services whose operations will be affected by your protocol. This is to ensure that prior to commencement of the investigation; these individuals have had an opportunity to assess the impact of the proposal on their area. This will include reviewing the proposed budget so they can accommodate any additional requirements arising from the protocol.

TITLE OF PROPOSED RESEARCH:
A Randomized Prospective Trial Comparing Pico-Salax (Magnesium Citrate) Plus Bisacodyl Versus Split Dose Polyethylene Glycol-Based Lavage In Preparation Of Patients For Colonoscopy

	Department Service	*Print Name*	*Signature*	*DATE*
☐ Yes ☐ No	Anesthesia			
☐ Yes ☐ No	Cardiac Diagnostics			
☐ Yes ☐ No	QIHI			
☐ Yes ☐ No	Diagnostic Imaging			
☐ Yes ☐ No	Health Records			
☐ Yes ☐ No	HMRC			
☐ Yes ☐ No	ICU			
☐ Yes ☐ No	Lab Med & Pathology			
☐ Yes ☐ No	Neurodiagnostics			
☐ Yes ☐ No	Nursing Unit			
☐ Yes ☐ No	Nursing Unit			
☐ Yes ☐ No	Nursing Unit			
☐ Yes ☐ No	Nutritional Services			
☐ Yes ☐ No	Outpatient Services			
☐ Yes ☐ No	Pharmacy			
☐ Yes ☐ No	Respiratory Therapy			
☐ Yes ☐ No	Surgical Services			
☐ Yes ☐ No	**Tumor Group Leader:TBCC**:			
☒ Yes ☐ No	Other: Gastroenterology			
☐ Yes ☐ No	Other:			
☐ Yes ☐ No	Other:			

My signature below acknowledges and accepts the impact (clinical, financial or otherwise) of this research study on my department/division/program/portfolio and I agree with the costs itemized in the study budget

______________________________ ______________________________ ____________
Signature of Department Head/Administrative Officer   Print Name   Date

Regulatory Approvals:

a) **Health Canada** TPD/NHP and Devices (this item applies only to studies involving drugs/devices or natural health products) Enrolment of subjects cannot start before the sooner of either receipt of the letter of no objection or the expiry of the period for Health Canada to object

Does the study involve off-label use of therapeutic agents, devices or natural health products? **No.**
If YES: Have you applied for a letter of no objection (LNO) from Health Canada Yes/No
If YES: Attach copy of LNO or indicate date of submission for HC review; (if NO: you will need to apply and have either a letter of no objection or expiry of the period for objection)

b) Stem Cell Research
Does the study involve stem cell research? Yes/**No**
If YES have you applied to the SCOC for review and approval?
Please attach letter of approval or indicate date of application

c) **University of Calgary**
Does the study involve items which may require review and certification by other university safety and compliance committees:

i. Institutional Biosafety Committee (Biohazards, Health and Safety)
ii. Animal Care (research animals)
iii. Environmental Health and Safety
iv. Radiation Safety Committee

If any of these apply, include a copy of the certificate with your application.

**SECTION C – RESEARCH PROTOCOL SUMMARY**<br>The purpose of this section is to outline the scientific structure of your study. Point form is acceptable.<br>All sections must be complete (do not simply refer to sections in your proposal).	
1. List a minimum of **3 key words** which describe your study (MeSH recommended). Please include keywords that would be interpretable by a non-medical audience<br>*For example: (1) Cardiology (2) hypertension (3) myocardial infarction*	
(1) Colonoscopy	(2) Polyps
(3) Preparation	(4) Colon Cancer
(5)	(6)
2. Does your study involve any of the following (check all that apply)?<br>☒ Questionnaire ☐ Interview ☐ Chart Review ☐ Database Linkage<br>Has this study received approval from a designated ethics committee in Alberta (i.e. CPSA, ACB, Uof A, Uof L, CREBA)?<br>☐ Yes (*If Yes, please append letter)*<br><br>☒ No<br>Has this study received science and ethics review and approval from a committee elsewhere in Canada?<br>☐ Yes (*If Yes, please append letter)* Please provide name of committee<br><br>☒ No	

3. Background Rationale:

The efficacy of colonoscopy in detecting abnormalities within the colon is highly dependent on the adequacy of the bowel preparation. Despite advances in bowel preparation, the process remains difficult for patients to tolerate and complete, and ultimately can result in missed lesions.[1] With published literature on missed polyps and carcinomas on colonoscopy,[2-4] the importance of optimal visualization of the colonic mucosa becomes more important. The increasing of knowledge of serrated adenomas, their propensity to progress to malignancy and their predilection for the right colon stresses the importance of adequate preparations.[67-70]

There has been a multitude of research into the optimal bowel preparation regimen[1, 5-65, 81-83] but the decision of which method to use remains largely in the hands of the individual colonoscopist. While some evidence suggested better efficacy with a 12 or 24 hour oral sodium phosphate (NaP) preparation over a whole dose polyethylene glycol (PEG) lavage, [66, 85] safety concerns of NaP still exist.[84-86, 71] In the past, oral NaP solutions have been favored by patients and physicians for their small volume. Significant electrolyte abnormalities can occur with NaP preparations and therefore limits their utility in hospitalized patients and those with significant cardiorenal comorbidities.[53, 62, 71-78, 84] NaP is no longer available over the counter in Canada, and therefore is now in limited use as a bowel preparation for colonoscopy. Polyethylene Glycol (PEG) Based Lavage is a widely used bowel preparation that has high efficacy for colon cleansing and a strong safety profile. [87] PEG preparations are relatively well tolerated from a taste perspective, but the high 4L volume is difficult to complete, [37] and therefore patient adherence to this regimen varies.

As an alternative, recent research has explored low-volume or split dose PEG preparations, sometimes with the addition of a stimulant laxative, in order to circumvent the issues surrounding preparation adherence in 4L doses, with positive results in regards to efficacy and tolerability[79, 89- 93]. Low-volume PEG plus ascorbic acid was as effective as a high-volume PEG plus electrolytes preparation, but had superior palatability [76, 81]. In a recent study conducted here at the Colon Cancer Screening Centre, a split dose (2L+2L) of Colyte, a PEG preparation, was found to provide a cleaner colon with less residual fluid than a one-time 4L dose of the same preparation, in both morning and afternoon procedures, although the best effect was seen when the last dose preceded the procedure by at least 5 hours.[94] Timing of split dose application has been outlined as an important factor in optimizing bowel cleansing and colon visualization.[61, 81, 94]

Pico-Salax (Sodium Picosulphate and Magnesium citrate) is a low-volume bowel preparation available in Canada. Evidence suggests that sodium picosulphate plus magnesium citrate bowel preparations, such as Pico-Salax, consistently yielded a high efficacy rating for colon cleansing[83] has been found to be at least as effective as PEG bowel preparations, [96] and may be much better tolerated, with fewer side effects.[79, 97] The addition of stimulant and osmotic laxatives to bowel preparations has shown increased efficacy in bowel cleansing [14, 16, 88, 91, 95, 98, 99]. In a recent study by Hookey, L. et al (2009)[82], Pico-Salax plus a two-day dose of Bisacodyl was found to provide superior colon cleansing in the right colon compared to Pico-Salax alone and NaP, and was found to be significantly better tolerated by patients than NaP. This is clinically significant as flat lesions are relatively more frequent in the right colon, and bowel preparation is often the poorest in this region[82]. The addition of Bisacodyl did not negatively affect the safety profile of Pico-Salax as a preparation. There is a lack of research on split dose Pico-Salax preparations; this study will examine the effect of splitting Pico-Salax dosing overnight in patients whose colonoscopy is occurring after 10AM.

4. Hypothesis/Research Question/Objectives

The objective of this study is to compare the efficacy, safety and tolerability of split dose Polyethylene Glycol-Based Lavage and Pico-Salax plus Bisacodyl with a specific emphasis on the right colon cleanliness.
The primary outcomes will be 1) quality of preparation in cleansing the colon, 2) quality of preparation in cleansing the right colon, 3) patient satisfaction. The secondary outcomes will be 1) duration of bowel preparation, 2) patient discomfort during bowel preparation.

5. Basic Study Design (Briefly describe your study):

All patients between the ages of 18 to 74 years referred to the Forzani Colorectal Cancer Screening Centre in Calgary, Alberta, Canada for colonoscopy will be considered for inclusion. During pre-assessments at the clinic, patients are asked to consider a general research consent. If they agree to that, then they will be approached for consideration of participating in this study and presented with an "Invitation to Participate in a Research Study" form (appendix); the study assistant will obtain final consent if they agree. Those not interested in participating will simply receive their physician's standard bowel preparation protocol. There will be no coercion of any sort. Enrollment of participants will be performed with block randomizations of 8 by using a computer-generated table, with allocation concealment maintained through the use of consecutively numbered sealed envelopes. Colonoscopists and investigators will be blinded to allocation groups. Patients will be allocated to one of two groups: (1) oral Pico-Salax (two sachets, with 1.5-2L of water following each sachet) with Dulcolax (Bisacodyl, 4 tablets); (2) split dose Polyethylene Glycol-Based Lavage (2L + 2L).

A study assistant will assign patients to their group and instruct them on the proper use of their assigned bowel preparation method. Patients will be given a tolerability questionnaire, which has been modified from a previously used questionnaire[94], to be completed once their bowel preparation is finished and before coming to the Centre for the colonoscopy (included in the appendix). Patient concerns or questions regarding the preparation will be directed toward the study assistant or clinic nurses as opposed to their endoscopist, so as to avoid unblinding the endoscopist.

6. Methods:

a) Subject Numbers: How many local subjects? 150 How many total subjects? 150

b) Major Inclusion/Exclusion Criteria:

Patients 18- 74 years referred to the Forzani Colorectal Cancer Screening Centre in Calgary, Alberta, Canada for colonoscopy will be considered. Major exclusion criteria will be acute coronary syndrome, congestive heart failure, unstable angina, known or suspected renal failure, ascites, megacolon, known or suspected bowel obstruction, or other comorbidities that may prevent colonoscopy as well as previous partial or subtotal colectomy or if the colonoscopy is warranted for the evaluation of diarrhea.

c) Interventions (What will be done to the subjects and for how long?)

Patients will be allocated to one of two groups: (1) oral Pico-Salax (two sachets, with 1.5-2L of water following each sachet) with Dulcolax (Bisacodyl, 4 tablets); (2) split dose Polyethylene Glycol-Based Lavage (2L + 2L). Specific instructions will be provided on preparation timing, depending on whether colonoscopy is prior to 10AM or after 10AM. Further instructions are given to participants regarding diet in the days leading up to their procedure (see appendix).

d) Primary Outcome Variables (Briefly describe what will be measured and how/using what measurement tools)

The previously validated Ottawa bowel preparation scale[80] will be used to assess the quality of bowel cleanliness. Each of the right, mid and rectosigmoid colon is rated on a 5-point scale (0-4). In addition, a complete 3-point rating for overall colonic fluid is assessed resulting in an overall score range of 0 to 14. An excellent preparation with little fluid would score 0 to 1; a good preparation, 2 to 4; while scores higher than 4 would indicate progressively worsening bowel preparations. A completely unprepared colon would score 11 to 14, depending on the amount of colonic fluid. Additionally a simplified overall cleanliness score will be utilized and compared to the Ottawa scale as a gold standard (both included in an appendix).

e) Limitations (please list briefly any limitations you have identified in the study)

7. Statistical Considerations in the study design:
   a) What is your sample size and how do you justify it? (Provide details of sample size calculation).

In our previous study at CCSC utilizing the Ottawa Prep score, we observed an effect size for segment scores that ranged from 0.5 to 1.0 (moderate) with a standard deviation of 1.02. For a two group ANOVA a total sample size of size of 130 patients will be required for a two tailed alpha at 0.05 and an 80% power. Based on the experience from our previous study, an additional 15%, or 20 patients, will be added to the total sample size to account for early withdrawals and incomplete colonoscopies.

   b) Data Analysis (Which method of analysis will you use?)

Descriptive statistics will be used for baseline characteristics. The Ottawa bowel prep scale produces data that is approximately normally distributed. Two group ANOVA will be used to assess for the presence of group differences in the prep scores between groups. The influence of colonoscopy time (AM vs PM) will be assessed by entering scope time as a factor in the ANOVA Model. The secondary endpoints of tolerability will be assessed using the Mann Whitney U test. A sub project will be to assess the agreement between the Ottawa Scale (validated gold standard) and the simplified overall score.

8. Recruitment:
   a) How will you identify potential subjects?

All patients aged 18-74 referred to the Forzani & MacPhail Colon Cancer Screening Centre in Calgary, Alberta will be considered for enrollment in the study.

   b) Who will recruit the potential subjects?

During pre-assessments at the clinic, patients will be asked to participate in the study by a study assistant. If they agree – final consent is obtained by the study assistant.

   c) Where will you recruit potential subjects?

We will recruit patients in the Colon Cancer Screening Centre that are referred for screening colonoscopy.

   d) What method (s) will you use to recruit potential subjects?

The study assistant, will approach potential patients and describe the study to them. Those not interested in participating will simply receive their physicians' standard bowel preparation protocol. There will be no coercion of any sort.

e) If your recruitment method involves a letter of invitation, poster, advertisement or electronic announcement, have you included a copy/copies in this application?

☒ Yes, included ☐ No ☐ N/A

f) Would you like an online poster for this study on the UofC website for public access? ☐ Yes ☒ No *(if yes, please contact the board office at 220-7990 for further details)*

APPLICATION FOR SCIENTIFIC, ADMINISTRATIVE & ETHICAL REVIEW OF HUMAN SUBJECT CLINICAL TRIALS AND HUMAN SUBJECT HEALTH RESEARCH
("Human Subject" includes human biosamples, tissue and genetic information as well as people)

SECTION D – BUDGET SUMMARY

To comply with regulatory requirements on the University of Calgary, full budget details are essential. YOUR APPLICATION CANNOT PROCEED WITHOUT BUDGETARY DETAILS. If your study is receiving funding from industry or a granting agency, you must attach a detailed budget. Any funding received from any external source is to be identified.

1. Human Resources (List all support personnel to be engaged in the study)

**Position**	**Salary**	**Benefits**	**Hrs/Wk**	**Total Hrs.**	**Cost**
Research Assistant	$24/ hr	+$3/hr		200	$ 5400.00<br>$<br>$<br>$<br>$
				Subtotal (1)	$

2. Service/Procedures (List x-rays ECGs, etc)

Service/Procedure	#Standard Care	#Research Specific	$Cost per item	Total
		X	$	= $
		X	$	= $
		X	$	= $
		X	$	= $
		X	$	= $
		X	$	= $
		X	$	= $

**Report tests which are usual care but do not include costs for these Tests in your calculations*

Subtotal (2) $

3. Supplies and Equipment (List all supply and equipment requirements)

Subtotal (3) $

4.Miscellaneous Items

Printing Costs

Subtotal (4) $ 500.00

5. Add-on costs: (Please list costs that will not be included in the final total, i.,e. start-up fee)

COST (Section 1-4)$ 5900.00

Industry Directed Studies Only: Overhead (25% applied to amt rec'd) $

REB Review Fee $

TOTAL COST (industry sponsored studies): $

FUNDING SOURCE(S): Complete sections A and B as applicable

A. **Non-industry Sponsored Funding:** List sources

Total Amt.	Status	
$	Applied	Received
$	Applied	Received
$	Applied	Received

Granting Agency **Internal (PI or Dept)** No Cost Other

B. Industry Directed Study: Grant <u>or</u> Contract

Company Name: ________________________________ Amount per patient $______________

Contact Person/Phone/Fax/Mail ____

1. Is overhead included in the amount per patient reported above? Yes No Not applicable
2. Will the local investigator or department receive additional funds for participating in this trial? Yes/No

ARE YOU REQUESTING FUNDS FROM CHR R&D? Yes No Amount requested $

SECTION E – PRIVACY PROTECTION

This section **MUST** be completed for all research studies. The ***Health Information Act*** requires an assessment of the risk to privacy. Please describe this below. Also describe how you will reduce the risk to privacy (see examples). A copy of the ***Health Information Act,*** is available at the public site for Canadian law: *canlii.org*

Project Privacy Management Issues

Please provide a response/details regarding all of these issues:

1. List all personal and health information sources and major data elements collected for study use.

Health information will be obtained from the patient and patient clinic chart.

2. For information source and major data element collected, describe purpose for collection and use of data (please relate purposes to information listed above).

The information collected will be used only for this study.

3. List the project personnel who have access to the information listed above.

Alaa Rostom – principal investigator
Robert Hilsden
Catherine Dube
Eleanor Rushton

4. Indicate whether any of the information will be disclosed to anyone other than project personnel, or for any purpose other than the purpose included in this application.

No

5. Does your project involve creating, reviewing or disclosing subjects' personal health information (eg. Health records, charts)? Yes ☐ No ☒

If your answer to 5, above, was " yes", the Health Information Act of Alberta requires you to obtain the subject's informed consent or to obtain a waiver of consent from the CHREB. (See CHREB policy and template documents on consent). The board's power to grant a waiver is highly circumscribed by law. The board can grant waivers only on grounds specified under the Health Information Act. If you need a waiver, you must frame your justification incorporating the terms set out in the Health Information Act (currently, section 50) See canlii.org
The only grounds currently for waiver are that it is unreasonable, impractical or not feasible to obtain consent. (Other grounds are not applicable.) It is up to applicants to provide reasons that fall under those headings.

EITHER (a) ATTACH PROPOSED CONSENT FORM(S)
OR (b) PROVIDE A JUSTIFICATION FOR A REQUEST FOR A WAIVER.

.

6. Describe the storage arrangements and final disposition of information collected for research purposes (include destruction dates).

All data will be kept in a locked cabinet. It will be kept for the duration of the study. It will be accessed by research personnel. The patient study file will only have their initials and a unique study number. A study key linking the patient number and names will be kept by the study coordinator in a locked drawer

7. Who has access to the information abstracted?
Research personnel only

8. Who has access to the listing of names and study ID numbers, if there is a study ID number?
Research personnel only

Privacy Risks and Controls Assessment	
Please provide an assessment of the privacy risks and controls used to mitigate these risks for project, including the following examples:	
Risk/Problem<br>Unauthorized external or internal access to identifying information through:<br>- Active use<br>- Transmission<br>- Storage<br>- Disposal	***Mitigation Measures/Solution***<br>- project personnel screening/agreements<br>- access authorization procedures<br>- designated systems administrator<br>- passwords/screen timeouts<br>- system access audits/disclosure logs<br>- secure mail/transport<br>- firewall/virus protect<br>- encrypted transmission<br>- secure paper-based storage<br>- shredding/wiping
Identification through publication or release	- Aggregations levels<br>- Alternate identifers
Identification through data-matching	Use of non-linkable elements or identifiers
Loss of data control outside jurisdiction	Confidentiality and security agreements for out-of-province recipients or storage providers
Loss of data control through non custodian contractors	Confidentiality and security agreements (e.g., information managers, ASPs)
Please provide an assessment of privacy risks and controls used to mitigate these risks for the project (you may use examples above if applicable):<br><br>All data will be kept in a locked cabinet. It will be kept for the duration of the study. It will be accessed by Research personnel. The patient study file will only have their initials and a unique study number. A study key linking the patient number and names will be kept by the study coordinator in a locked drawer	

**SECTION F – BIOGRAPHICAL SKETCH OF PRINICIPAL INVESTIGATOR**
☐ A recent CV (within 3 years of the date of this application) is on file at Child Health Research Office, or the Office of Medical Bioethics.<br>**OR**<br>☒ I have included one copy of my CV with this application (current CVs may be submitted electronically to chreb@ucalgary.ca).
